# Air pollution associated acute respiratory inflammation and modification by GSTM1 and GSTT1 gene polymorphisms: a panel study of healthy undergraduates

**DOI:** 10.1186/s12940-022-00954-9

**Published:** 2023-01-27

**Authors:** Xiang Zeng, Ge Tian, Jingfang Zhu, Fuyun Yang, Rui Zhang, Huijun Li, Zhen An, Juan Li, Jie Song, Jing Jiang, Dongling Liu, Weidong Wu

**Affiliations:** 1 Henan International Collaborative Laboratory for Health Effects and Intervention of Air Pollution, School of Public Health, Xinxiang Medical University, 601 Jinsui Road, Xinxiang, Henan, 453003 China; 2 School of Public Health, Zhejiang Chinese Medical University, 548 Binwen Road, Hangzhou, 310053 Zhejiang Province China; 3 School of Basic Medical Science, Zhejiang Chinese Medical University, 548 Binwen Road, Hangzhou, 310053 Zhejiang Province China

**Keywords:** Air pollution, Air pollutants, Inflammation, Oxidative stress, Lung function, Glutathione S-Transferase

## Abstract

Epidemiological evidence has linked air pollution with adverse respiratory outcomes, but the mechanisms underlying susceptibility to air pollution remain unclear. This study aimed to investigate the role of glutathione S-transferase (GST) polymorphism in the association between air pollution and lung function levels. A total of 75 healthy young volunteers aged 18–20 years old were recruited for six follow-up visits and examinations. Spirometry was conducted to obtain lung function parameters such as forced vital capacity (FVC), and forced expiratory volume in 1 s (FEV_1_). Nasal fluid concentrations of interleukin-6 (IL-6), interleukin-8 (IL-8), tumor necrosis factor-α (TNF-α), and 8-epi-prostaglandin F2α (8-epi-PGF2a) were measured using ELISA kits. Linear mixed-effect models were used to evaluate the association of air pollutants with respiratory outcomes. Additionally, polymorphisms of glutathione S-transferase mu 1 (GSTM1) and glutathione S-transferase theta 1 (GSTT1) were estimated to explore its role in the association between air pollutants and lung function. We found that short-term exposure to atmospheric particulates such as PM_2.5_ and PM_10_ can cause an increase in nasal biomarkers of inflammation, oxidative stress, and lung function, while air gaseous pollutant exposure is linked with decreased lung function, except for CO. Stratification analyses showed that an increase in nasal inflammatory cytokines caused by exposure to atmospheric particulates is more obvious in subjects with *GSTM1*-sufficient (*GSTM1*^+^) than *GSTM1*-null (*GSTM1*^*−*^), while elevated lung function levels due to air particles are more significant in subjects with the genotype of *GSTM1*^*−*^ when compared to *GSTM1*^+^. As for air gaseous pollutants, decreased lung function levels caused by O_3_, SO_2_, and NO_2_ exposure is more manifest in subjects with the genotype of *GSTM1*^*−*^ compared to *GSTM1*^+^. Taken together, short-term exposure to air pollutants is associated with alterations in nasal biomarkers and lung function levels in young healthy adults, and susceptible genotypes play an important mediation role in the association between exposure to air pollutants and inflammation, oxidative stress, and lung function levels.

**Supplementary Information:**

The online version contains supplementary material available at 10.1186/s12940-022-00954-9.

## Introduction

Air pollution is a global problem that has overtaken other environmental risks to become the fourth overall risk factor for death and the first major environmental risk factor due to the economy's rapid growth and the steady promotion of urbanization. According to the Global Burden of Disease Study 2019, air pollution is responsible for seven million deaths worldwide, or 11.75% of all fatalities [1]. Air pollution is complicated and characterized by high concentrations of fine particulate matter (PM_2.5_) and ozone (O_3_) in China because of rising coal consumption, automobile ownership, and industrial emissions [2]. Due to its small size, PM_2.5_ can enter the lung through the ciliated airway, and some of it can also the blood–brain barrier and enter the blood circulation [3]. Haze, characterized by a higher level of PM_2.5_, has a wide range of impacts, including making life, travel and work more difficult or inconvenience, as well as having a negative impact on public health [4–6]. Respiratory health is most affected by air pollution because the respiratory system is the first line of the body to contact and resist air pollutants.

Air pollution can decrease lung function and increase the risk of pathogenesis or exacerbation of respiratory diseases such as asthma, chronic obstructive pulmonary disease (COPD), pneumonia, and lung cancer [7–17]. However, genetic susceptibility to air pollutants like PM_2.5_, coarser particulate matter (PM_10_), O_3_, sulfur dioxide (SO_2_), nitrogen dioxide (NO_2_) and carbon monoxide (CO) varies from person to person [18–20]. Inflammation and oxidative stress are, according to previous research, the primary underlying cause of respiratory damage caused by air pollution. [21–23]. Glutathione S-transferases (GSTs) have been implicated in the maintenance of cell integrity, defense against oxidative stress and DNA damage, and detoxification of endogenous and exogenous compounds in the cell [24]. The absence of bioactive antioxidant protein expression in the GSTT1 null (*GSTT1*^*−*^) and GSTM1 null (*GSTM1*^*−*^) genotypes reduces the body's antioxidant capacity [25]. The prevalence of *GSTM1*-null and *GSTT1-null* polymorphisms across ethnic groups varied from 18 to 66% and 10% to 58%, respectively [26–28].

There were fewer epidemiological studies investigating the respiratory effects of air pollution exposure between different genotypes of *GSTM1* and *GSTT1*, and the existing research results were inconsistent [29]. For example, Gilliland et al. displayed that children with *GSTM1*^*−*^ and *GSTP1*^*−*^ genotypes have higher lung function levels such as FVC and FEV_1_ when compared to their peers with *GSTM1*^+^ and *GSTP1*^+^ [30]. He et al. indicated that smokers with GST null genotypes have a more obvious decline in lung function than GST sufficient genotypes [31]. Imboden et al. found that there was an accelerating decline in lung function in the general male population with genetic *GSTT1* deficiency, but not females [22, 32], reported that the frequency of incense burning at home increased the risk of current asthma and exercise wheeze among children with *GSTT1*^−^ genotype [33]. However, several studies demonstrated that there were no significant associations between GST variation and respiratory outcomes [34–36]. Therefore, more optimized research is needed to further confirm the mediation role of susceptible genotypes in air pollution and human health.

In this panel study, 75 undergraduates from Xinxiang Medical University between the ages of 18 and 21 were recruited to investigate the influence of exposure to air pollutants on acute respiratory inflammation and the underlying mechanisms based on inflammatory biomarkers like interleukin-6 (IL-6); interleukin-8 (IL-8); fibroblast growth factor (FGF-); as well as 8-epi-prostaglandin F2 (8-epi-PGF2); Additionally, the genotype strata were administered in order to evaluate the potential modifying effect of GSTM1- and GSTT1-null genotypes on associations between exposure to air pollution and adverse respiratory effects.

## Materials and methods

### Study design and participant recruitment

**Fig. 1:**
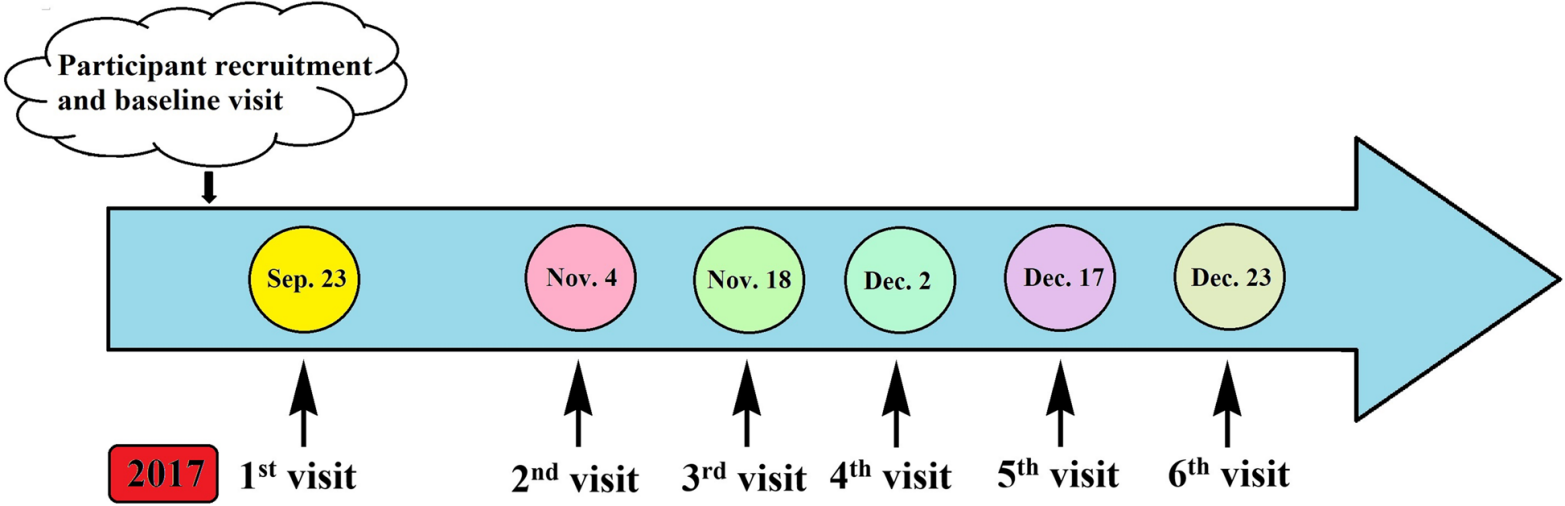
The flow chart of this panel study

We scheduled a longitudinal panel study among freshmen from Xinxiang Medical University in Central China from September 23 to December 23 with six follow-ups lasting at least one week (Fig. 1). Before the follow-up study, a baseline survey was done, and questionnaires were used to get information about each individual's gender, age, weight, height, smoking and alcohol use, health, cardiovascular, and respiratory histories through face to face interview. Exposure levels of air pollutants were consecutively monitored for 7 days before the physical examination. Thee inclusion criteria of the participant’s health status were no smoking, no drinking, no cardiovascular and respiratory symptoms or diseases, and other chronic disorders. To reduce heterogeneity in lifestyle, dietary habits, and concurrent exposure to other pollutants, we excluded seven participants who lived off campus. Finally, this study included 75 participants who met the study's eligibility requirements. All participants agreed and completed their written informed consent after receiving detailed explanations of the study and potential consequences before enrollment. This study protocol was approved by the Human Ethical Committee of Xinxiang Medical University, China (NO. 2017–02-0623).

### Exposure measurements

The air pollution exposure data were obtained from a real-time data acquisition and automatic quality control system of air monitoring stations (Thermo Fisher Scientific Co., Ltd., Waltham, MA, USA), which was installed on the rooftop of a building with seven floors (about 21 m high) on the campus of Xinxiang Medical University. This site is close to the dorms, playgrounds, and classrooms of the participants at a distance smaller than 1000 m. Hourly PM_2.5_, PM_10_, O_3_, SO_2_, NO_2_, and CO concentrations (24-h average concentration) were measured before the day of health measurements, and meteorological data including temperature (T) and relative humidity (RH) were collected at the same time. Personal PM_2.5_ and PM_10_ sample exposure concentrations were calculated based on each physical examination time. The air quality complex index (AQCI) was calculated by the equation of AQCI = ∑ C_i_/S_i_, where C_i_ is the concentration of air pollutants including PM_2.5_, PM_10_, O_3_, SO_2_, NO_2_, and CO; S_i_ is the annual mean level II of the national ambient air quality standard of China (NAAQS-China). The average daily dose (ADD) of air pollutants for individuals was further evaluated based on the equation of ADD = AQCI × [inhalation rate/weight] [37].

### Health measurements and lab analyses

Physical examination was conducted using an automatic digital height and weight measuring instrument (Omron HBF-371, Kyoto, Japan), and more detailed measurement information as described in our previous study [38]. Spirometry was performed using an electronic spirometer following the standardized procedures of the American Thoracic Society (ATS)-criteria (Chestgraph HI-801, CHEST Ltd., Tokyo, Japan). Flow-volume curve manoeuvers and volume-time curves of lung function parameters such as forced vital capacity (FVC), forced expiratory volume in 1 s (FEV_1_), the ratio of FEV_1_ to FVC (FEV_1_/FVC), peak expiratory flow (PEF), forced expiratory flow at 25% of FVC (FEF_25_), forced expiratory flow at 75% of FVC (FEF_75_), maximal voluntary ventilation (MVV), and minute ventilation (MV) were measured three times, and the highest value of lung function parameters (the best test performance) was used in the analysis [39].

Commercially available assay kits were used to measure inflammatory biomarkers like IL-6, IL-8, and TNF-, as well as oxidative stress indicators like 8-epi-PGF2 in nasal epithelial lining fluid (ELF). Each participant had two nostrils sprayed with approximately 100 L of sterile normal saline, and their noses were kneaded until they were completely moist. To obtain nasal EFLs, the test paper was then inserted into the two nostrils with a length that was appropriate for the nasal cavity [40]. After being clamped for two minutes, the test strips were taken out, placed on a clean worktable with ventilation, and dried for 24 h at room temperature. Then, the test strips were eluted using the elution buffer composed of 1% BSA, 0.05% Triton X-100, and Dulbecco's PBS with 100 μLper strip. The supernatant was stored at -20℃ after centrifugation for 2 min at 13,000 rpm [19]. IL-6, IL-8, TNF-α, and 8-epi-PGF2α were measured with the corresponding ELISA Kits according to the guidelines of the manufacturers.

A total of 2 mL venous blood was collected from each participant into a vacuum blood collection tube. Genomic DNA was extracted from peripheral blood lymphocytes using a QIAamp DNA Blood Mini Kit (Qiagen, Valencia, CA, USA). Polymorphisms of *GSTM1* and *GSTT1* were conducted using multiplex polymerase chain reaction (PCR) amplification. The primers of *GSTM1* and *GSTT1* were 5’-GAACTCCCTGAAAAGCTAAAGC-3’ (Forward) and 5’-GTTGGGCTCAAATATACGGTGG-3’ (Reverse), and 5’- TTCCTTACTGGTCCTCACATCTC-3’ (Forward) and 5’-TCACCG GATCATGGCCAGCA-3’ (Reverse), respectively (Table S1).

### Statistical analyses

Linear mixed-effects models with a random intercept for each participant were used to evaluate the associations between air pollutants and health outcome biomarkers, which allow each subject to serve as his or her own control over time and has the advantage of explaining correlations among multiple repeated measurements collected per participant including a random intercept for each subject [19]. Health outcome data were log-transformed to improve normality and matched with air pollution data before the nasal epithelial lining fluid collection. Two different sets of exposure metrics (lag 0 to lag 6 and 1-d to 7-d moving averages) were used to identify the most relevant exposure metric(s), which may best capture the associations between air pollution and health indicators [41]. The single-constituent model was used to estimate the consistency of the examined association of specific air pollutants and biomarkers. The linear mixed-effects model was utilized to assess the mixed effect of air pollutants including PM_2.5_, PM_10_, O_3_, SO_2_, NO_2_, and CO on biomarkers such as IL-6, IL-8, TNF-α, and 8-epi-PGF2α in the current study. Air pollutant concentrations and health outcome data were merged by physical examination date. A series of potential confounders including age, body mass index, time trend, day-of-week, study location, temperature, and relative humidity were adjusted in the statistical models [42]. Results were demonstrated as estimated percent changes with 95% confidence intervals (CIs) in biomarkers associated with interquartile range (IQR) increases in air pollutants. All analyses were conducted using software SAS 9.2 (SAS Institute, Cary, NC, USA), and the significant level was set at *p* < 0.05 (2-tailed).

## Results

### Descriptive statistics of exposure and health measurements

A total of 75 volunteers participated in the physical examination. Air pollutants, blood samples, and nasal ELF were repeatedly collected and analyzed six times. The demographic characteristics of the 75 participants are shown in Table 1. There were 21 males and 54 females with an average age of 18.61 ± 0.72 years and a mean BMI of 20.84 ± 2.89 kg/m^2^. Among 75 volunteers, 35 and 38 subjects had *GSTM1*-sufficient (*GSTM1*^+^) and *GSTT1*-sufficient (*GSTT1*^+^) genes, respectively. The percentage of *GSTM1*-null (*GSTM1*^*−*^) and *GSTT1*-null (*GSTT1*^*−*^) was 53.3% and 49.3%, respectively. There was no significant difference between GST subgroup genotypes in age, sex, and BMI (Table 1).

**Table 1:** Descriptive characteristics of 75 participants in this study

	GSTM			GSTT			Total (*n* = 75)
	GSTM1^+^ (*n* = 35)	GSTM1^−^ (*n* = 40)	p-value	GSTT1^+^ (*n* = 38)	GSTT1^−^ (*n* = 37)	p-value	
Age (years)	18.69 ± 0.72	18.54 ± 0.72	0.382	18.43 ± 0.73	18.68 ± 0.67	0.463	18.61 ± 0.72
Height (cm)	162.87 ± 8.90	165.67 ± 6.28	0.126	163.86 ± 0.07	165.42 ± 0.08	0.389	164.38 ± 7.74
Weight (kg)	54.00 (47.00 ~ 59.00)	56.35 (51.25 ~ 59.50)	0.421	55.00 (50.00 ~ 59.25)	55.00 (50.00 ~ 59.50)	0.858	55.00 (50.00 ~ 59.25)
BMI (kg/m^2^)	20.83 ± 3.31	20.84 ± 2.47	0.985	20.87 ± 2.66	20.68 ± 3.11	0.781	20.84 ± 2.89
Sex [male, n (%)]	10 (28.60)	11 (27.50)	0.972	11 (28.95)	10 (27.03)	0.797	21 (28.0)

*Abbreviations*: *BMI* body mass index, *GSTM1* gene of glutathione S-transferase mu 1, *GSTT1* gene of glutathione S-transferase theta 1, *GSTM1*^+^ GSTM1-sufficient, *GSTM1* GSTM1-null, *GSTT1*^+^ GSTT1-sufficient, GSTT1 GSTT1-null

The health characteristics of the participants are demonstrated in Table 2. The range of the concentrations of air pollutants was manifested as follows: PM_2.5_ (33.0 to 401.0 µg/m^3^), PM_10_ (22.0 to 369.0 µg/m^3^), O_3_ (00.0 to 200.0 µg/m^3^), SO_2_ (16.0 to 113.0 µg/m^3^), NO_2_ (14.0 to 119.0 µg/m^3^), and CO (0.8 to 6.0 mg/m^3^) during the period of the six visit time points (Table 2). The alterations in the concentration of air pollutants over time were inconsistent. ADD of air pollutants for undergraduates is 2.915 ± 0.399, and there is no significant difference in ADD and related subgroups between males and females (Table S2).

**Table 2:** Daily ambient air pollutants, biomarkers, PM_2.5_-bound heavy metal composition, lung function, and meteorological parameters during the period of the panel study

	Each follow-up time levels (median)						Total follow-up period levels			
	T1	T2	T3	T4	T5	T6	min	Median (25^th^,75^th^)	max	IQR
Air pollutants										
PM_2.5_ (μg/m^3^)	278.0	66.0	138.5	149.5	88.0	186.5	33.0	132.5 (86.5,198.5)	401.0	112.0
PM_10_ (μg/m^3^)	173.0	65.0	224.5	183.0	93.5	206.5	22.0	148.0 (88.5, 215.5)	369.0	127.0
O_3_ (μg/m^3^)	56.0	30.5	38.0	11.0	10.5	3.5	0	17.5 (4.5, 48.0)	200.0	43.5
SO_2_ (μg/m^3^)	25.5	19.5	25.0	34.0	31.5	74.0	16.0	31.0 (23.0, 39.5)	113.0	16.5
NO_2_ (μg/m^3^)	66.0	50.5	43.5	68.5	50.5	88.0	14.0	57.0 (40.5, 71.0)	119.0	30.5
CO (mg/m^3^)	2.6	1.9	2.4	2.9	3.0	4.6	0.8	2.6 (2.2, 2.6)	6.0	0.85
Lung function levels										
FVC (L)	3.03	3.13	3.10	3.11	3.07	3.05	2.01	3.09 (2.79, 3.62)	5.39	0.83
FEV1 (L)	2.62	2.80	2.87	2.82	2.79	2.75	1.31	2.78 (2.53, 3.24)	5.01	0.71
FEV_1_/FVC (%)	87.10	92.65	92.91	92.50	92.26	90.53	47.46	90.92 (85.02, 95.65)	100	10.63
FEF_25_ (L/s)	3.92	4.76	4.76	5.10	4.79	4.60	0.55	4.51 (3.24, 5.41)	9.04	2.17
FEF_75_ (L/s)	2.03	2.24	2.34	2.38	2.25	2.24	0.87	2.43 (1.91, 3.03)	8.33	1.12
PEF (L/s)	4.13	4.92	5.00	5.27	4.90	4.94	1.04	4.83 (3.89, 5.69)	9.52	1.80
MVV (L/min)	69.20	79.15	88.40	87.95	87.65	92.35	14.30	84.65 (69.22, 105.95)	175.00	36.73
MV (L/min)	8.17	8.57	9.05	9.53	8.75	9.18	2.04	8.82 (7.07, 11.18)	28.06	4.11
Biomarkers										
IL-6 (pg/mL)	6.3	17.7	33.4	9.2	12.6	52.0	0.1	22.10 (3.53, 54.10)	852.2	50.57
IL-8 (pg/mL)	39691.9	21792.6	41135.9	17431.7	23894.9	36387.8	213.0	28944.3 (11372.2, 56421.2)	154813.1	45049.0
TNF-α (pg/mL)	26.0	10.0	19.7	29.8	87.7	62.4	0.2	44.51 (12.87, 87.75)	522.0	74.88
8-epi-PGF2a (pg/mL)	83638.3	71937.5	51730.8	47157.1	56559.0	91455.5	670.7	64942.9 (28634.1, 107913.1)	193830.7	79279.0
Weather										
Temperature (℃)	27.0	12.9	7.7	4.8	1.6	6.6	-2.0	6.8 (4.0, 12.9)	30.5	8.9
Relative humidity (%)	61.0	54.0	30.5	54.5	44.5	50.0	19.0	50.0 (41.0, 56.0)	84.0	15.0

*Abbreviations*: Forced vital capacity (FVC), forced expiratory volume in 1 s (FEV_1_), the ratio of FEV_1_ to FVC (FEV_1_/FVC), peak expiratory flow (PEF), forced expiratory flow at 25% of FVC (FEF_25_), forced expiratory flow at 75% of FVC (FEF_75_), maximal voluntary ventilation (MVV), and minute ventilation (MV); fine particulate matter (PM_2.5_), coarser particulate matter (PM_10_), ozone (O_3_), sulfur dioxide (SO_2_), nitrogen dioxide (NO_2_) and carbon monoxide (CO); interleukin-6 (IL-6); interleukin-8 (IL-8); tumor necrosis factor (TNF-α); and 8-epi-prostaglandin F2α (8-epi-PGF2α)

The average levels of lung function parameters including FVC, FEV_1_, FEV_1_/FVC, PEF, FEF_25_, FEF_75_, MVV, and MV were 3.09 L, 2.78 L, 90.83, 4.51 L/s, 2.43 L/s, 4.83 L/s, 84.65 L/min, and 8.82 L/min, respectively. Mean biomarker levels of IL-6, IL-8, TNF-α, and 8-epi-PGF2α in nasal ELF were 22.10 ng/mL, 28,944.30 ng/L, 44.51 pg/L, and 64,714.32, respectively. During the period of the study, the average concentrations of air pollutants including PM_2.5_, PM_10_, O_3_, SO_2_, NO_2_, and CO were 132.5 μg/m^3^, 148.0 μg/m^3^, 17.5 μg/m^3^, 31.0 μg/m^3^, 57.0 μg/m^3^, and 2.60 mg/m^3^, respectively. The average temperature and relative humidity were 6.8℃ and 50.0% during the period of this study, respectively.

### Relationship between air pollutants and inflammatory mediators and lung function

Spearman correlation analyses were used to investigate the correlation between air pollutants, inflammatory and oxidative stress biomarkers, and lung function parameters.

Air pollutants such as PM_2.5_, PM_10_, NO_2_, SO_2_, and CO were positively correlated with each other except for O_3_ (Table 3). Additionally, ADD and related ADD_PM2.5_, ADD_PM10_, ADD_SO2_, ADD_NO2_, ADD_O3_, and ADD_CO_ was significantly negatively correlated with FVC and FEV_1_. Moreover, the direction of correlation between each air pollutant and nasal biomarkers was not completely inconsistent in this study. However, there was no significant correlation between ADD and nasal biomarkers such as IL-6, IL-8, TNF-α, and 8-epi-PGF2a. Interestingly, nasal inflammatory and oxidative stress biomarkers, such as IL-6, IL-8, TNF-α, and 8-epi-PGF2a, were significantly positively correlated with FVC and FEV_1_ (Table 3).

**Table 3:** Spearman correlation analyses of the relationship between air pollutants, inflammatory cytokines, and lung function

	PM_2.5_	PM_10_	O_3_	SO_2_	NO_2_	CO	T	RH	IL-6	IL-8	TNF-α	8-epi-PGF2α	FVC	FEV_1_	FEV_1_/FVC	PEF	FEF_25_	FEF_75_	MVV	MV
PM_2.5_	—																			
PM_10_	**0.598**	—																		
O_3_	**-0.234**	**-0.267**	—																	
SO_2_	**0.134**	**0.232**	**-0.130**	—																
NO_2_	**0.488**	**0.515**	**-0.647**	**0.406**	—															
CO	**0.479**	**0.403**	**-0.495**	**0.588**	**0.639**	—														
T	**0.228**	**0.076**	**0.462**	**-0.136**	**-0.068**	**-0.345**	—													
RH	**0.667**	**-0.344**	**-0.542**	**-0.054**	**0.453**	**0.535**	**-0.113**	—												
IL-6	**-0.196**	-0.008	-0.059	**0.292**	**-0.101**	**0.306**	-**0.148**	**-0.300**	—											
IL-8	0.045	0.025	0.032	**0.094**	**-0.137**	0.046	0.031	-0.081	**0.270**	—										
TNF-α	**-0.345**	**-0.351**	**-0.293**	-0.075	**-0.427**	**-0.105**	**-0.422**	**-0.349**	**0.315**	**0.380**	—									
8-epi-PGF2α	-0.042	-0.037	0.020	0.075	-0.076	0.072	-0.019	**-0.102**	**0.199**	**0.917**	**0.374**	—								
FVC	0.040	0.042	0.015	-0.013	0.063	-0.002	0.043	0.045	**0.171**	**0.251**	**0.263**	**0.308**	—							
FEV_1_	-0.028	0.047	-0.087	-0.004	0.089^#^	0.040	-0.039	-0.031	**0.201**	**0.285**	**0.251**	**0.307**	**0.852**	—						
FEV_1_/FVC	**-0.126**	-0.001	**-0.192**	-0.013	0.064	0.056	**-0.149**	**-0.127**	0.043	**-0.120**	-0.073	-0.083	**-0.359**	0.075	—					
PEF	**-0.177**	-0.008	**-0.198**	0.060	0.031	**0.120**	**-0.191**	**-0.179**	**0.130**	**0.119**	**0.159**	**0.150**	**0.440**	**0.604**	**0.368**	—				
FEF_25_	-**0.405**	-0.056	**-0.429**	**0.097**	0.078	**0.248**	**-0.420**	**-0.401**	**0.168**	0.062	**0.162**	**0.127**	**0.429**	**0.629**	**0.426**	**0.972**	—			
FEF_75_	**0.386**	**0.113**	**0.336**	**-0.100**	-0.003	**-0.222**	**0.379**	**0.384**	-0.030	0.022	-0.062	-0.032	0.054	**0.408**	**0.765**	**0.456**	**0.506**	—		
MVV	**-0.242**	-0.037	**-0.213**	**0.127**	-0.035	**0.174**	**-0.249**	**-0.204**	**0.210**	**0.150**	**0.264**	**0.149**	**0.653**	**0.722**	0.050	**0.600**	**0.593**	**0.328**	—	
MV	-**0.140**	-0.007	**-0.096**	**0.102**	0.004	**0.129**	**-0.130**	**-0.128**	**0.233**	0.041	0.062	0.031	**0.224**^**#**^	**0.238**	0.016	0.181	0.174	0.039	**0.262**	—

*Abbreviation*: PM_2.5_ fine particulate matter, *PM*_*10*_ coarser particulate matter, *O*_*3*_ ozone, *SO*_*2*_ sulfur dioxide, *NO*_*2*_ nitrogen dioxide, *CO* carbon monoxide, *T* temperature, *RH* relative humidity, *Al* aluminum, *Ba* barium, *Sr* strontium, *V* vanadium, *Cr* chromium, *Mn* manganese, *Ni* nickel, *Cu* copper, *Zn* zinc, *Cd* cadmium, *Pd* palladium, *Ge* germanium, *As* arsenic, *B* boron, *Pb* lead

The statistically significant correlations are highlighted in bold

### Association and modification of GST polymorphisms effects of air pollutants on nasal biomarkers and lung function in undergraduates

Short-term exposure to atmospheric particulates such as PM_2.5_ and PM_10_ can cause an increase in nasal biomarkers of inflammatory and oxidative stress and lung function levels, whereas air gaseous pollutant exposure linked with alteration of nasal biomarkers of inflammatory and oxidative stress and decreased lung function levels, except for CO. Specifically, atmospheric particulate exposure can significantly increase the levels of IL-6 and TNF-α with the characteristics of accumulation with exposure days. For example, each IQR (112 μg/m^3^) increase in PM_2.5_ was associated with an increase in TNF-α by 81.57%, and each IQR (127 μg/m^3^) increase in PM_10_ was linked with an increase in IL-6 by 190.77%. In addition, each 10 μg/m^3^ increase in PM_2.5_ were associated with increased FVC by 0.75% (95% confidence interval [CI]: 0.12%, 1.37%) at lag 7; each 10 μg/m^3^ increase in PM_10_ was associated with increased FVC by 2.36% (1.35%, 3.37%) at lag 5. Moreover, a 10 μg/m^3^ increase in PM_10_ was associated with a 1.41% (95%CI: 0.37%, 2.44%) increment in PEF and a 1.39% (0.32%, 2.46%) increment in MVV, respectively. However, each 10 μg/m^3^ increase in O_3_ was associated with reduced FVC by 15.61% (-25.10%, -6.13%) at lag 3. Short-term exposure to CO caused bidirectional changes in lung function parameters such as FVC, PEF, and MVV. In particular, CO exposure can lead to an increase in lung function at lag 1–4 days and subsequently result in a decline in lung function at lag 5–7 days (Fig. 3, Fig. S3, Fig. S4). In brief, there were different effects of particulate pollutants and gaseous pollutants in the atmosphere on the level of nasal biomarkers and lung function of undergraduates.

**Fig. 2:**
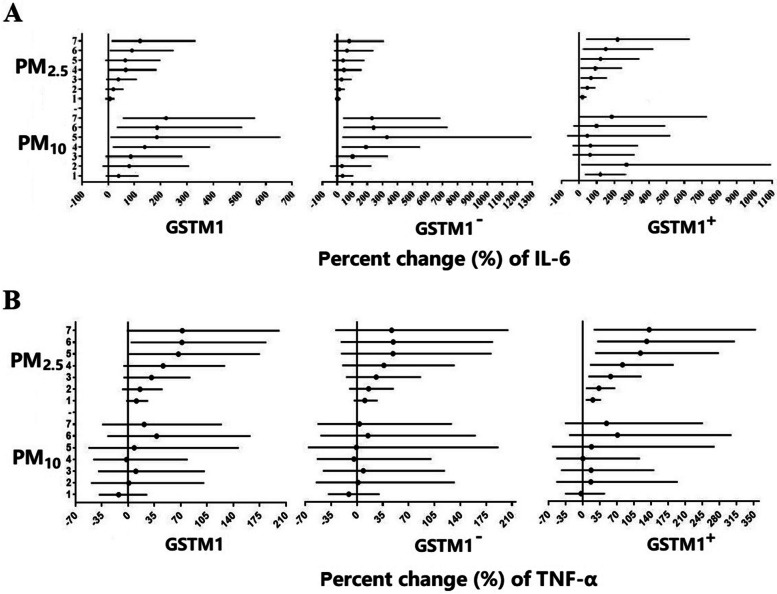
Changes in GST modified effects of the estimated percent changes with 95% confidence intervals in interleukin-6 (IL-6) and tumor necrosis factor (TNF-α) associated with air particulate pollutants from 1-day to 7-day averages

**Fig. 3:**
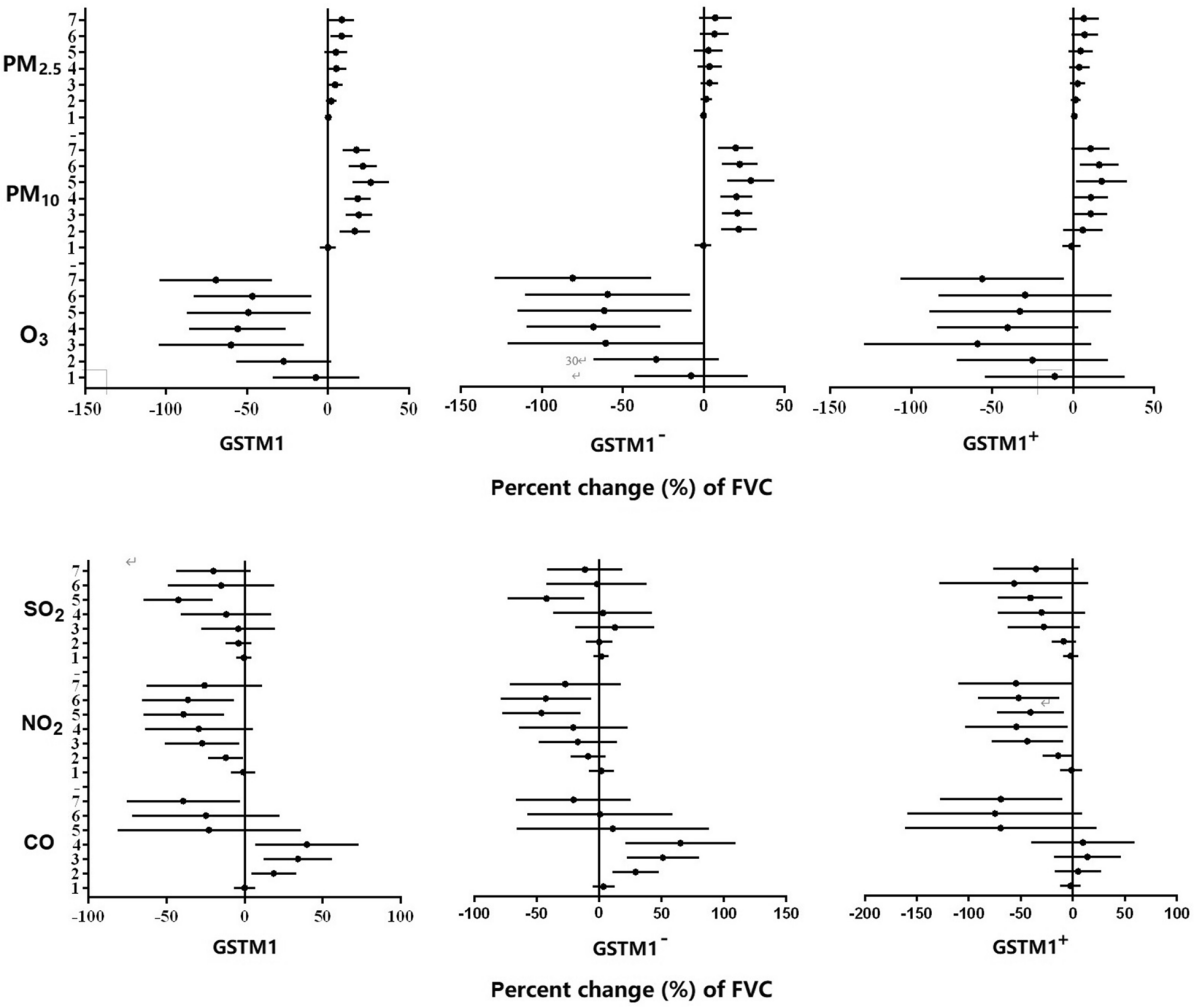
Changes in GST modified effects of the estimated percent changes with 95% confidence intervals in forced vital capacity (FVC) associated with air pollutants from 1-day to 7-day averages

Stratification analyses allow us to have a clearer understanding of the health response of susceptibility genes with different genotypes when exposed to air pollutants. There is a positive trend between PM_2.5_ and percent change of IL-6 in the full *GSTM1* population, and the trend is more pronounced in subjects with *GSTM1*^+^ genotype as PM_2.5_ exposure accumulates (Fig. 2A). Similarly, the concentration of TNF-α increases with the accumulation of PM_2.5_ exposure, and the trend is more manifest in subjects with (Fig. 2B). In addition, both nasal IL-8 and 8- epi-PGF2a demonstrated a consistently increasing trend as exposure to airborne particulates increases, although it is not statistically significant (Fig. S1). Notably, atmospheric gaseous pollutants did not show a good consistent trend with nasal biomarkers of inflammation and oxidative stress no matter what the *GSTM1* genotype (data not shown). Alteration in lung function change is different when exposure to particulate pollutants and gaseous pollutants, and in the specific *GSTM1* genotype. Specifically, exposure to PM_2.5_ and PM_10_ is associated with elevated lung function parameters such as FVC, PEF, and MVV in the full *GSTM1* population, and the trend is more obvious in subjects with *GSTM1*^*−*^ genotype as particulate exposure accumulates (Fig. 3, Fig. S2-S4). Exposure to O_3_, NO_2_, and SO_2_ leads to a reduction in lung function levels in the full *GSTM1* population, although it is not statistically significant. It is worth mentioning that the above-mentioned trend is more visible in the subjects with *GSTM1*^*−*^ genotype.

## Discussion

Through a panel study of healthy undergraduates, this study investigated the association between exposure to major air pollutants, biomarkers of airway inflammation, and lung function. The influence of GST polymorphism modification on the effects of air pollutants on lung function was further investigated. Our results indicated that short-term particulate exposure including PM_2.5_ and PM_10_ was associated with an increase in IL-6, TNF-α, and lung function. However, exposure to gaseous pollutants including O_3_, SO_2_, and NO_2_ was associated with a decrease in lung function including FVC, FEV_1_, PEF, and MVV. In general, lung function levels are lower in subjects with the *GSTM1*^*−*^ genotype than in those with the *GSTM1*^+^ genotype. A plethora of studies has investigated the influence of air pollutant exposure on lung function. However, the relationship between air pollution and lung function has been inconsistent. There was an inverse association between PM_2.5_ exposure and lung function according to most of the previous studies. For example, a recent study showed that short-term personal exposure to PM_2.5_ was linked with reduced spirometer parameters such as FVC and FEV_1_ of old adults from the South of China [43]. Similarly, short-term personal exposure to NO_2_ was associated with an increase of respiratory inflammation parameter as fractional exhaled nitric oxide (FeNO) and a decline in lung function levels such as FVC, FEV_1_ and PEF [44]. An areview indicated that an increase in outdoor air particulate pollutant exposure was associated with a decrease in FEV_1_ in healthy adults [45]. In addition, another study pointed out that short-term personal exposure to PM_2.5_ or ultrafine particles were not associated with lung function of old adults in the Netherlands, Switzerland, and Italy [46]. The results may be attributed to the low PM_2.5_ exposure levels of approximately to 15 μg/m^3^ in the above-mentioned studies. Additionally, a repeatedly-measured study with 34 healthy nonsmoking adult volunteers showed that short-term exposures to particulate matter were associated with slightly increased levels of FEV_1_ and FEF_25-75_ [47]. Another panel study demonstrated that a short-term averaging time (3-d moving average) air pollutant exposure was positively associated with lung function, while a longer averaging time (14-d moving average) air pollutant exposure was negatively associated with lung function [48].

In line with previous studies, there was an inverse relationship between lung function and atmospheric gaseous pollutants like O_3_, SO_2_, NO_2_, and CO [49]. However, we found a positive association between young healthy undergraduates' lung function and short-term exposure to PM_2.5_ or PM_10_, which was consistent with some previous studies [47,48]. The estimated effects of particulate matter on lung function are not only dependent on its exposure concentration and compositions but also influenced by characteristics of the exposure population such as age, genetics, and basic health status. In this study, particulate such as PM_2.5_ and PM_10_ mainly deposits on the upper respiratory tract and may not cause serious damage to the lower respiratory tract. In addition, the subjects are healthy young adults, and their bodies will produce compensatory and adaptive responses to particulate matter, which may explain the positive relationship between exposure to particulate matter and lung function in this study. Gaseous pollutants such as O_3_, SO_2_, NO_2_, and CO can be directly dissolved in the lower respiratory tract such as alveoli and bronchioles, leading to severe lung impairment, such as lower lung function even in healthy young adults.

Previous epidemiological studies have indicated that the underlying mechanisms of inflammation and oxidative stress play a critical role in driving the adverse respiratory effects of particulate matter. For example, PM_2.5_ exposure has been associated with elevated levels of inflammatory biomarkers including IL-6, IL-8, TNF-α, and FeNO, and oxidative stress biomarkers such as 8-epi-PGF2α [50–52], which is consistent with our results of atmospheric particulate exposure in the current study. A recent meta-analysis of 22 epidemiological studies reported that exposure to PM_2.5_ is associated with an increased level of IL-6 [53]. Another meta-analysis of 23 epidemiological studies summarized that PM_2.5_ exposure may contribute to increased levels of oxidative stress biomarkers such as malondialdehyde (MDA), superoxide dismutase (SOD), and 8-hydroxy-2'-deoxyguanosine (8-OHdG) [54]. In the current study, we found that exposure to PM_2.5_ and PM_10_ was associated with increased levels of nasal inflammatory and oxidative stress biomarkers such as IL-6, IL-8, TNF-α, and 8-epi-PGF2α.

There are numerous studies investigating the relationship between atmospheric gaseous pollutants such as O_3_, SO_2_, NO_2_, and CO and biomarkers of inflammation and oxidative stress including IL-6, IL-8, TNF-α, 8-hydroxy-2'-deoxyguanosine (8-OHdG), and 8-epi-PGF2α. For instance, a panel study of young healthy students demonstrated that O_3_ and SO_2_ are the two major traffic-related pollutants positively associated with biomarkers of inflammation and oxidative stress such as high-sensitivity C-reactive protein (hs-CRP), fibrinogen and 8-OHdG [55]. Average 2-week O_3_ exposure was significantly and positively associated with IL-1β, IL-8, IL-17A, IFN-γ, and TNF-α in healthy adults [56]. An increase in NO_2_ exposure was associated with elevated levels of IL-6 as well as IL-10 in 8-year-old children [57]. In addition, there was a negative correlation between SO_2_, NO_2_, or CO and fibrinogen in the male population [58]. Short-term exposure to NO_2_ and O_3_ and long-term exposure to CO were inversely associated with fibrinogen in Korean elderly adults [59]. However, there were a lot of studies with no significant results between other atmospheric gaseous pollutants not mentioned above and biomarkers of inflammation and oxidative stress [21, 56, 57]. In the present study, we found that there was no significant association between gaseous pollutants and inflammatory biomarkers such as IL-6, IL-8, TNF-α, and oxidative stress biomarker 8-epi-PGF2α in nasal epithelial lining fluid, which may be due to the short retention time and the lower absorption concentration of gaseous pollutants in the nasal cavity.

Previous studies have provided substantial evidence showing gene-air pollution interactions for cardiovascular outcomes [60]. However, the interplay of air pollution and specific genes related to detoxification (*GSTM1* and *GSTT1*) on respiratory health is still lacking and unclear. This study demonstrated that the genetic susceptibility of *GSTM1* and *GSTT1* played a crucial role in influencing air pollution exposure on lung function. *GSTM1*-null (*GSTM1*^*−*^) and *GSTT1*-null (*GSTT1*^*−*^) individuals are more susceptible to air pollution exposure such as PM_2.5_ and PM_10,_ distinguishing levels of biomarkers and lung function due to the lack of corresponding detoxifying enzymes when compared with their *GSTM1*-sufficient (*GSTM1*^+^) and *GSTT1*- sufficient (*GSTT1*^+^) peers, respectively. Deletion polymorphisms of the GST gene have an additive effect of air pollution on nasal mucus biomarkers and lung function. Previous studies confirmed that *GST* polymorphisms can modify the effect of air pollution on biomarkers and health outcomes [61–63]. Notably, the actual phenotype of health outcomes is determined by various factors such as heredity, environment, basic constitution, underlying health conditions, and their interactions.

This study has a few limitations. First, personal exposure levels of air pollutants derived from the fixed monitoring station may not reflect the actual exposure of each subject and lead to an underestimation of the influence of air pollutants. Second, due to the closer correlations among air pollutants, we did not run two pollutant models to distinguish the independent influence of each pollutant. Third, a large sample is a necessary condition for accurate estimation, and effect estimation accuracy may not be achieved with the small sample size. Fourth, other potential confounders may act because there are a lot of unmeasured air pollutants that might link with biomarkers and lung function. Finally, we did not estimate the interactive impact of other antioxidant enzyme genes with GST gene polymorphisms and air pollutants.

## Conclusions

In summary, this study suggests that short-term exposure to air pollutants alters nasal biomarkers of inflammation and oxidative stress and lung function levels in young healthy adults. Nasal mucus sampling is a novel method worth popularizing for evaluating the effects of atmospheric pollutant exposure on respiratory health outcomes. *GSTM1* genotypes play an important mediation role in the association between exposure to air pollutants and inflammation, oxidative stress, and lung function levels. This study may shed light on the mechanism of *GST*-related genotype and subsequent influence on human health.

## Supplementary Information

**Additional file 1.**
