# Supplementary material for: Air pollution associated acute respiratory inflammation and modification by GSTM1 and GSTT1 gene polymorphisms: a panel study of healthy undergraduates
Source: Environ Health. 2023 Jan 27;22:14. doi: 10.1186/s12940-022-00954-9 (PMC9881318; doi:10.1186/s12940-022-00954-9)
Supplement: Supplementary file 1 — Additional file 1. [file 12940_2022_954_MOESM1_ESM.docx]

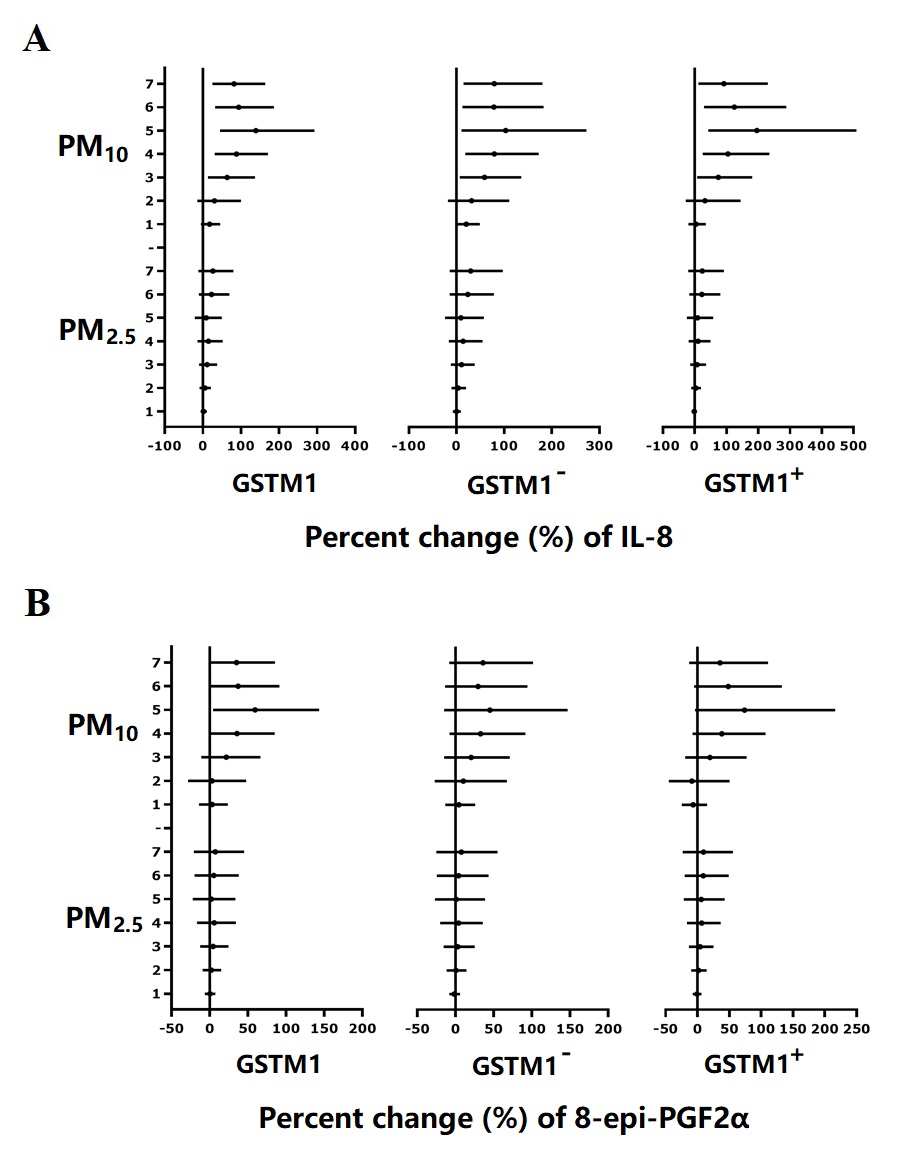


**Fig. S1.** Changes in GST modiﬁed eﬀects of the estimated percent changes with 95% conﬁdence intervals in interleukin-8 (IL-8, Fig. S1A) and 8-epi-prostaglandin F2α (8-epi-PGF2a, Fig. S1B) associated with air pollutants from 1-day to 7-day averages.


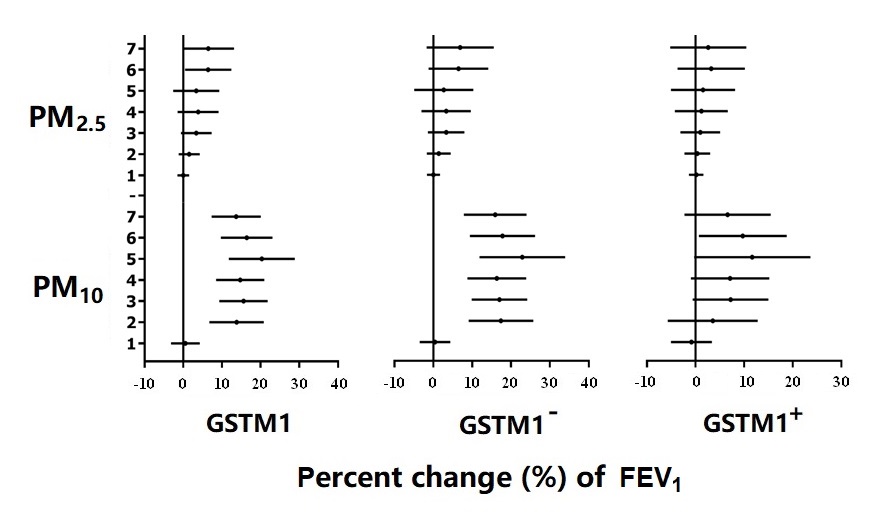


**Fig. S2.** Changes in GST modiﬁed eﬀects of the estimated percent changes with 95% conﬁdence intervals in forced expiratory volume in 1 s (FEV_1_) associated with air pollutants from 1-day to 7-day average.


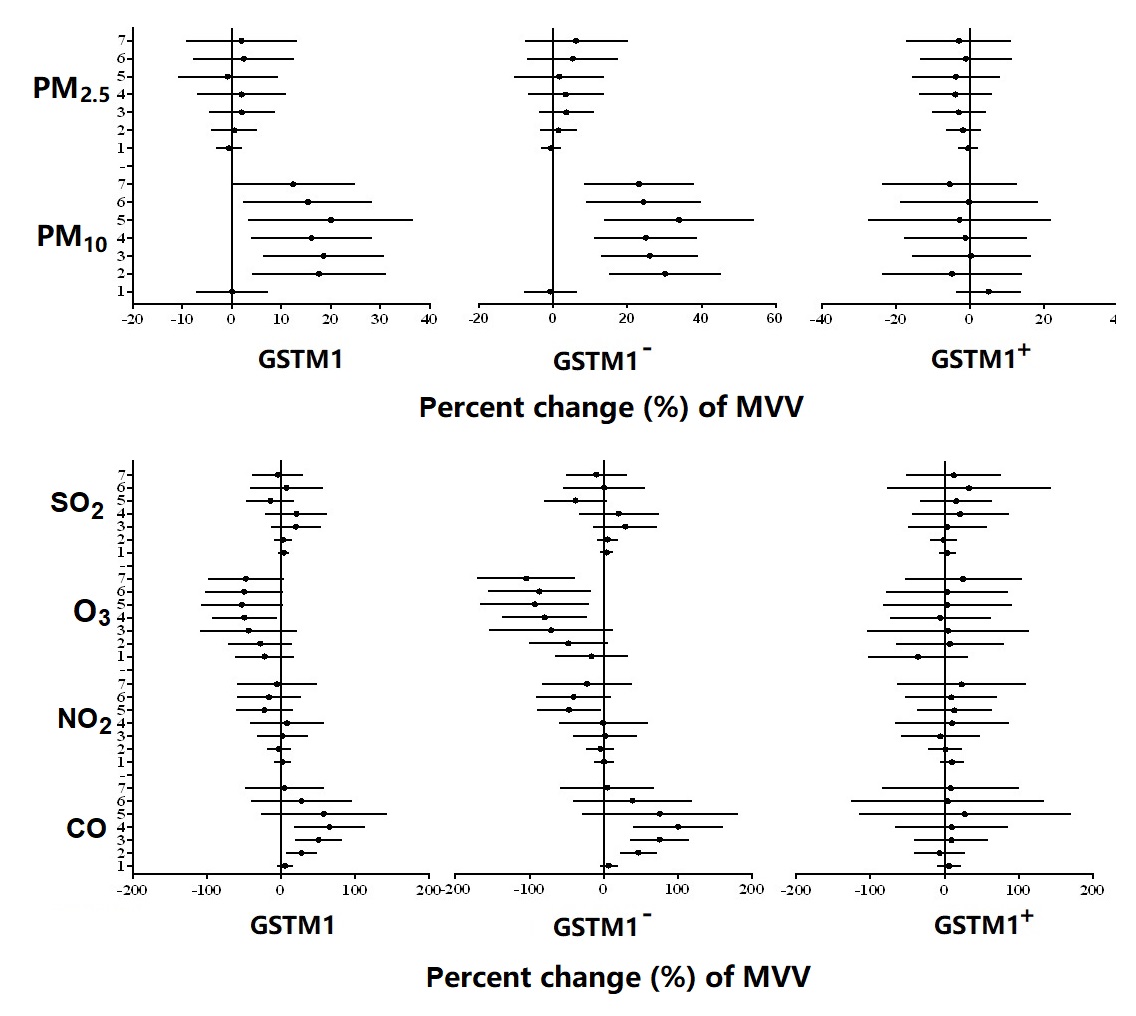


**Fig. S3.** Changes in GST modiﬁed eﬀects of the estimated percent changes with 95% conﬁdence intervals in maximal voluntary ventilation (MVV) associated with air pollutants from 1-day to 7-day average.


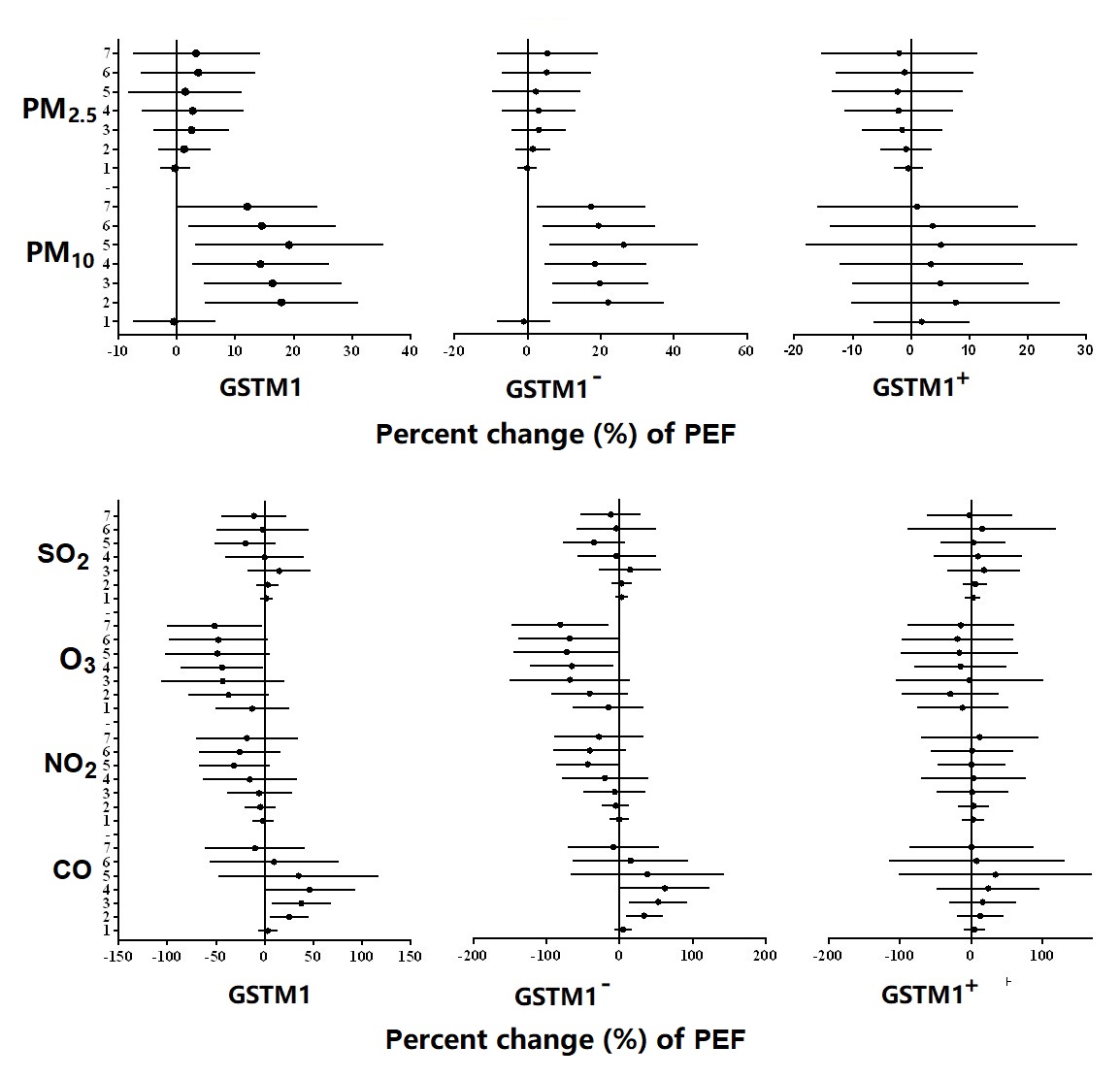


**Fig. S4.** Changes in GST modiﬁed eﬀects of the estimated percent changes with 95% conﬁdence intervals in peak expiratory flow (PEF) associated with air pollutants from 1-day to 7-day average.

**Table S1.** Amplification primers for the *GSTM1* and *GSTT1* genes.

| Primer name | Sequence |
| --- | --- |
| *GSTM1* | Forward: 5’-GAACTCCCTGAAAAGCTAAAGC-3’ |
|  | Reverse: 5’-GTTGGGCTCAAATATACGGTGG-3’ |
| *GSTT1* | Forward: 5’-TTCCTTACTGGTCCTCACATCTC-3’ |
|  | Reverse: 5’-TCACCGGATCATGGCCAGCA-3’ |

**Table S2.** The average daily dose (ADD) of air pollutants for individuals.

| ADD | Levels | | P value |
| --- | --- | --- | --- |
|  | Male | Female |  |
| ADD_Full_ | 2.778 ± 0.535 | 2.969 ± 0.320 | 0.063 |
| ADD_PM2.5_ | 1.223 ± 0.235 | 1.307 ± 0.141 |  |
| ADD_PM10_ | 0.683 ± 0.131 | 0.730 ± 0.079 |  |
| ADD_SO2_ | 0.167 ± 0.032 | 0.178 ± 0.019 |  |
| ADD_NO2_ | 0.460 ± 0.089 | 0.492 ± 0.053 |  |
| ADD_O3_ | 0.035 ± 0.007 | 0.038 ± 0.004 |  |
| ADD_CO_ | 0.210 ± 0.040 | 0.224 ± 0.024 |  |

Abbreviation, ADD: average daily dose; ADD_PM2.5_: average daily dose of PM_2.5_; ADD_PM10_: average daily dose of PM_10_; ADD_SO2_: average daily dose of SO_2_; ADD_NO2_: average daily dose of NO_2_; ADD_O3_: average daily dose of O_3_; ADD_CO_: average daily dose of CO; PM_2.5_: particulate matter with particle size below 2.5 microns; PM_10_: Particulate matter with particle size below 10 microns; SO_2_: sulfur dioxide; NO_2_: Nitrogen dioxide; O_3_: ozone; CO: carbon monoxide.
